# Synthesis, Molecular Modeling and Biological Evaluation of Metabolically Stable Analogues of the Endogenous Fatty Acid Amide Palmitoylethanolamide

**DOI:** 10.3390/ijms21239074

**Published:** 2020-11-28

**Authors:** Alessia D’Aloia, Federica Arrigoni, Renata Tisi, Alessandro Palmioli, Michela Ceriani, Valentina Artusa, Cristina Airoldi, Giuseppe Zampella, Barbara Costa, Laura Cipolla

**Affiliations:** 1 Department of Biotechnology and Biosciences, University of Milano-Bicocca, Piazza della Scienza 2, 20126 Milano, Italy; alessia.daloia@unimib.it (A.D.); federica.arrigoni@unimib.it (F.A.); renata.tisi@unimib.it (R.T.); alessandro.palmioli@unimib.it (A.P.); michela.ceriani@unimib.it (M.C.); v.artusa@campus.unimib.it (V.A.); cristina.airoldi@unimib.it (C.A.); giuseppe.zampella@unimib.it (G.Z.); 2 Milan Center for Neuroscience (NeuroMI), University of Milano-Bicocca, P.zza dell’Ateneo Nuovo 1, 20126 Milano, Italy

**Keywords:** palmitoylethanolamide, fatty acid amide hydrolase, inflammation, PPAR-α receptor, metabolism, PEA analogues

## Abstract

Palmitoylethanolamide (PEA) belongs to the class of *N*-acylethanolamine and is an endogenous lipid potentially useful in a wide range of therapeutic areas; products containing PEA are licensed for use in humans as a nutraceutical, a food supplement, or food for medical purposes for its analgesic and anti-inflammatory properties demonstrating efficacy and tolerability. However, the exogenously administered PEA is rapidly inactivated; in this process, fatty acid amide hydrolase (FAAH) plays a key role both in hepatic metabolism and in intracellular degradation. So, the aim of the present study was the design and synthesis of PEA analogues that are more resistant to FAAH-mediated hydrolysis. A small library of PEA analogues was designed and tested by molecular docking and density functional theory calculations to find the more stable analogue. The computational investigation identified RePEA as the best candidate in terms of both synthetic accessibility and metabolic stability to FAAH-mediated hydrolysis. The selected compound was synthesized and assayed ex vivo to monitor FAAH-mediated hydrolysis and to confirm its anti-inflammatory properties. ^1^H-NMR spectroscopy performed on membrane samples containing FAAH in integral membrane protein demonstrated that RePEA is not processed by FAAH, in contrast with PEA. Moreover, RePEA retains PEA’s ability to inhibit LPS-induced cytokine release in both murine N9 microglial cells and human PMA-THP-1 cells.

## 1. Introduction

Fatty acid ethanolamides are a family of endogenous bioactive compounds abundant in the central nervous system, attracting great attention due to their physiological, pro-homeostatic, and therapeutic potential for the treatment of various pathological conditions, such as inflammation, neurodegenerative diseases, and neuropathic pain. Among this class of compounds, palmitoylethanolamide (PEA, *N*-(2-Hydroxyethyl)hexadecanamide) is particularly interesting. PEA acts through different mechanisms affecting multiple pathways, both at the cellular and molecular level; however, arising evidence is demonstrating that one of the few identified mechanisms for anti-inflammatory PEA effects is due to peroxisome proliferator-activated receptor-α (PPAR-α) binding, resulting in the activation of PPAR-α-dependent gene transcription [1].

The main drawback of fatty acid ethanolamides as therapeutics is their poor in vivo metabolic stability, due to their fast hydrolysis by a series of hydrolytic enzymes, such as fatty acid amide hydrolase (FAAH), *N*-acylethanolamine acid amidase (NAAA), and monoacylglycerol lipase (MAGL). Concerning exogenous PEA as a drug, few data on its pharmacokinetics in humans or experimental animals are currently available; this issue has been recently reviewed by Rankin and Fowler who stated that “concerning the ADME of PEA there are large gaps in our knowledge” [2]. However, these data suggest that PEA produces limited systemic exposure levels, with plasma concentrations remaining in the nM range and with significant increases only for a short period; oral administration of PEA leads to a variable increase in plasma concentration, ranging from 2- to 9-fold from baseline [3]. The administration of 300 mg of ultramicronized PEA to healthy volunteers doubled the plasma level at 2 h with undetectable levels at 4 h [4]. FAAH-mediated metabolism may be, at least in part, responsible for the limited exposure of exogenous PEA. In fact, hydrolytic enzymes participate not only in the regulation of specific tissue levels of PEA but also in the first-pass effect, since the liver is the second organ, after the brain, in which FAAH shows the highest specific activity [5]. For these reasons, in the last decades a great deal of research has been focused on the design of ethanolamide analogues with increased stability to hydrolysis [5,6].

The key issue to ethanolamide analogues is maintaining the higher stability of hydrolytic enzymes while preserving the ability to bind to responsive receptors, such as PPAR-α. In this work, we propose the design of a small library of PEA analogues and an in silico study of PPAR-α ligands with hydrolytic stability towards FAAH and NAAA. The most promising compound was synthesized and preliminary biological evaluation is reported.

## 2. Results

### 2.1. Design of PEA-Analogues

**Figure 1:**
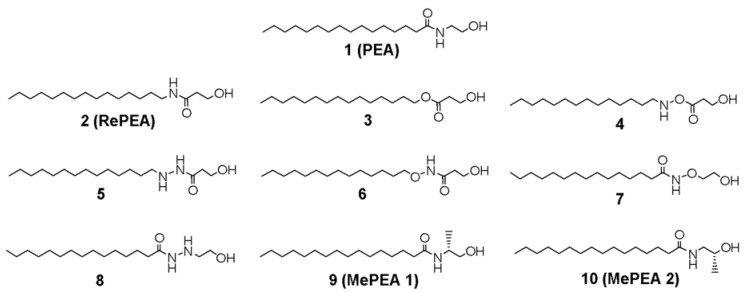
Chemical structures of PEA (palmitoylethanolamide) and its analogues.

PEA is a structurally simple amide constituted of palmitic acid as the acid component and the two carbon 2-aminoethanol as the amine moiety. Besides the amido group, the only additional functionality is the hydroxyl group, representing the polar head of the molecule. The aim of this work is to design PEA analogues that are still able to maintain biological activity and which possess longer life in vivo than their natural counterpart and are more stable to the hydrolytic action of FAAH. Both FAAH and NAAA exert their catalytic activity through a first nucleophilic attack of catalytic serine or cysteine, respectively, to the carbonyl group (vide infra); this first step towards hydrolysis may be influenced by stereoelectronic effects. Variation in the carbonyl group substituents can modify their propensity towards the nucleophilic attack of the catalytic amino acid, as well as steric hindrance. In order to modify carbonyl electrophilicity, modifications of the amidic bond are proposed: at first, the isosteric analogue **2** (RePEA, Figure 1), possessing a retroamide linkage between pentadecanamine and 2-hydroxypropanoic acid, was considered. In order to design PEA analogues with potentially increased stability, the amide bond was also substituted with an ester bond (**3**, Figure 1), with alkoxy amines, either with the alkoxy moiety as a mimic of the 2-aminoethanol polar head (compounds **4** and **7**, Figure 1) or as the apolar tail (**6**, Figure 1), and with acyl hydrazides (**5** and **8**, Figure 1). Finally, in order to evaluate the steric effect both on hydrolysis and PPAR-α affinity, two commercially available PEA analogues were also considered, (*R*)-palmitoyl-(1-methyl) ethanolamide (**9**, also referred to as MePEA1, Figure 1), which reproduces the presence of a methyl group similar to methanandamide [6] and (*R*)-palmitoyl-(2-methyl) ethanolamide (**10**, also referred to as MePEA2, Figure 1), where the methyl group is located on the *β*-carbon from the amidic group.

### 2.2. Analogues of PEA Can Equally Bind PPAR-α Receptor

**Figure 2:**
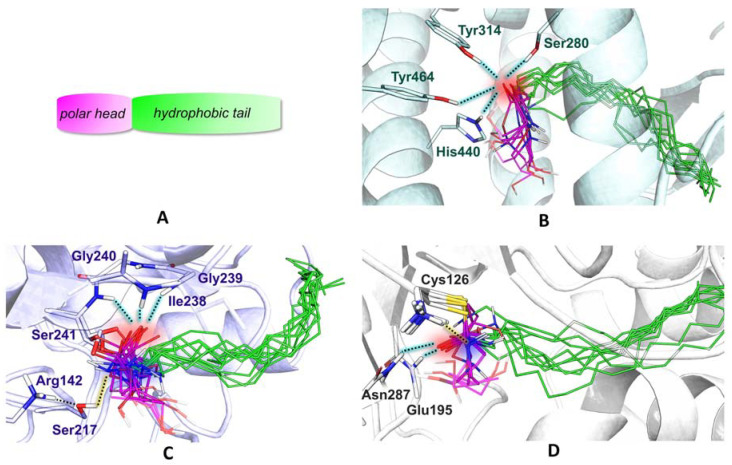
(**A**) Schematic scaffold of PEA and analogues **2**−**10**. They all feature a polar head, which is represented in pink, and a hydrophobic tail, in green. (**B**) Docking poses of PEA and analogues **2**–**10** into the PPAR-α (peroxisome proliferator-activated receptor-α) pocket. The side chains of key residues, Ser280, Tyr314, Tyr464, and His440 are shown as sticks. (**C**) Covalent docking poses of PEA and analogues **2**–**10** into the FAAH (fatty acid amide hydrolase) active site. All ligands are covalently bound to Ser241. The rest of the catalytic triad, formed by Ser217 and Arg142, together with the oxyanion hole, formed by Ser241, Gly240, Gly239, and Ile238, are shown as sticks. (**D**) Covalent docking poses of PEA and analogues **2**–**10** into the NAAA active site. All ligands are covalently bound to Cys126. The oxyanion hole, formed by Asn287 and Glu195, is highlighted with stick representation. Oxygen, nitrogen, and sulfur atoms are colored in red, dark blue, and yellow, respectively. Significative non-bonding interactions are represented as black dotted lines. The ones involving the carbonyl group of ligands are highlighted in light blue, whereas the ones involving the amine group of ligands are highlighted in yellow. The different conformations of Ser241 (in (**C**)) and Cys126 (in (**D**)) result from covalent docking simulations, which imply geometry relaxation of the reactive residue, upon covalent bond formation.

In the attempt of preserving the biological activity of PEA analogues, we took advantage of the availability of the X-ray crystal resolved PPAR-α receptor ligand-binding domain structure. All the ligands of the library have been designed purposefully to maintain the general PEA molecular scaffold (Figure 2A) to minimize the effect on yet uncharacterized sites of action. Molecular docking of PEA and its analogues to the binding domain of PPAR-α revealed a very similar binding mode (Figure 2B). Interestingly, even the top XP Glide scores of all ligands are rather close in energy, in a range from −6.0 to −8.4 kcal/mol.

The polar head and lipidic tail of each ligand are superimposed, occupying the arm I and arm II of the PPAR-α Y-shaped cavity, respectively (Supplementary Figure S1). This feature is essentially shared by most known PPAR full agonists [7,8]. Arm I is the only substantially polar cavity of PPAR-α and it extends toward the C-terminal helix12, referred to as AF2 (activation function-2) helix. The involvement of the latter in a conserved hydrogen bonds pattern has been proposed to be at the basis of PPAR agonism [9]. Particularly, the interaction between Tyr464 and the ligands has been found to be crucial for regulating the co-activator recruitment. Such an interaction, together with other H-bonds involving three polar residues that are highly conserved in the arm I of each PPAR isotype (Ser280, Tyr314, and His440 in PPAR-α) [10], has been proposed to hold the AF2 helix in the active conformation which is permissive for interactions with co-activators [11].

Remarkably, we found that these key interactions are intercepted by PEA and all of the docked analogues (Figure 2B). Indeed, Ser280, His440, and Tyr314 interact with the carbonyl group of the ligand amide bond, while in the lower energy poses Tyr464 interacts invariably with the carbonyl functionality or with the hydroxyl group of the ethanolamine portion.

**Figure 3:**
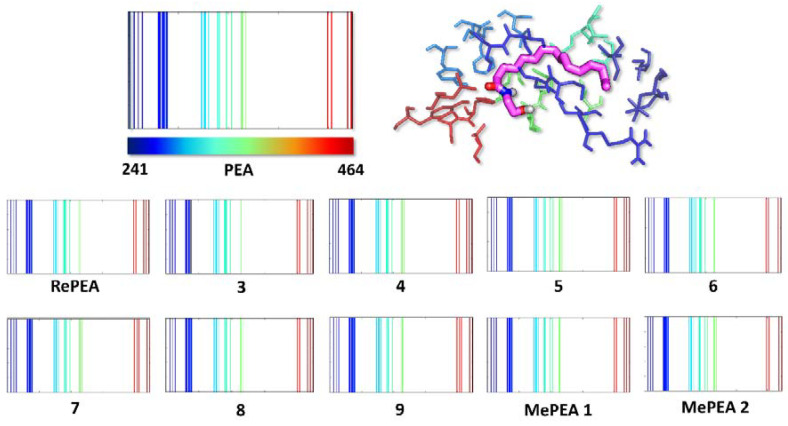
Structural Interactions Fingerprints (SIFs) of PEA and its analogues within the PPAR-α receptor. Each ligand is represented by a binary string encoding its interactions with the receptor residues, that are found between Ile241 and Tyr464. A solid-colored line in the SIFs indicates that the ligand is involved in one (or more) interaction(s) with the residue of the corresponding color (from dark blue to dark red).

In order to further assess the similarity of the designed ligands with PEA, in terms of their interaction with PPAR-α, we built Structural Interaction Fingerprints (SIFs), which capture the 3D information associated with a receptor–ligand complex into a 1D representation. This tool, generally used for docking-based virtual screening and poses clustering [12], allowed us to straightforwardly compare the interaction patterns provided by our docked ligands within the PPAR-α pocket (Figure 3). The SIFs obtained, considering the lowest energy pose of each compound, are all almost identical.

This observation means that all the designed ligands interact with PPAR-α in a strictly analogous fashion, i.e., the same portion of each ligand generates essentially the same interactions with the same proximal residue(s). Since all the ligands also show a very similar predicted affinity for the receptor, we should expect, at least in principle, the triggering of the same biological response, at least as far as PPAR-α is involved. Moreover, the conservation of structural and molecular properties of PEA in all of the designed analogues suggests that they should retain interaction with other PEA targets as well.

### 2.3. All of the Designed Compounds Are Eligible as FAAH Substrate

Once verified that any structural modifications rationally introduced to PEA scaffold should not alter biological activity, we performed molecular docking on FAAH, to analyze the behavior of the ligands toward FAAH-mediated hydrolysis. Once more, covalent docking results suggest that PEA and its analogues should be similarly accommodated within the catalytic pocket (Figure 2C). In particular, their polar head directly interacts with the catalytic triad formed by Ser241, Ser217, and Lys142, while the hydrophobic chain occupies the acyl-chain binding pocket (ABP) [13]. This conformation should represent the pre-reactive pose for ligand hydrolysis [14,15]. According to the reported catalytic mechanism, the catalytic triad assists the proton transfer from Ser241 to the unprotonated Lys142, mediated by Ser217. Ser241 is thus responsible for the nucleophilic attack to the C=O bond of the ligand and the formed tetrahedral intermediate is stabilized by the oxyanion hole constituted by Ser241, Gly240, Gly239, and Ile238 [16,17]. Interestingly, the ligand interactions with the oxyanion hole are found in all of the best poses of each ligand, suggesting that they should undergo hydrolysis according to a general mechanism that is similar to the typical one proposed for fatty acid ethanolamides, such as PEA (see Supplementary Figure S2 for ligand-specific ligand interaction diagrams and scores). Other conserved interactions found in the docked ligand–protein complexes involve Ser217 and Met191, in line with previous investigations [18]. In particular, Ser217 interacts with the ligand amine functionality, prompted by the subsequent proton transfer that assists in leaving group release (ethanolamine in the case of PEA). The amine moiety is also involved in a H-bond interaction with the backbone of Met191. The latter is also found to interact through its sidechain with the hydroxyl head of some docked ligand. The CovDock results do not provide any structural or energetic rationale of hypothetical different stability of the docked ligands toward hydrolysis.

### 2.4. Identification of Analogues with a Higher Metabolic Stability

**Figure 4:**
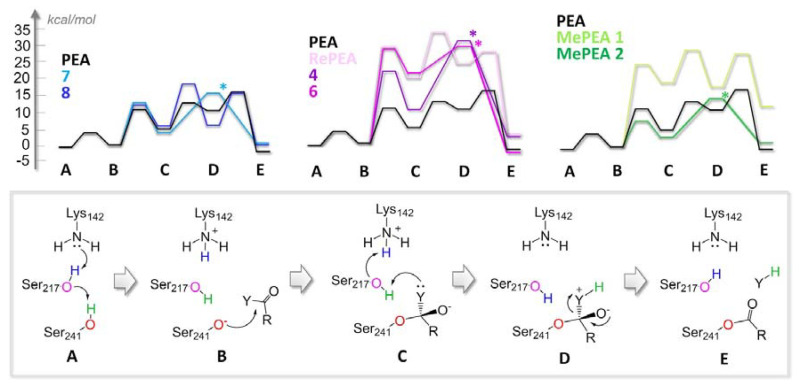
Top: energy profiles (kcal/mol) associated with the FAAH acylation phase of hydrolysis of PEA-like ligands (left blue shades), RePEA-like ligands (middle, purple shades), and MePEA ligands (right, green shades). The PEA hydrolysis profile (black lines) has been taken as a reference for each plot. If present, “*” indicates a concerted elementary reaction step from C to E (i.e., without passing through the formation of D. Bottom: reaction mechanism corresponding to the calculated energy profiles.

The structural variability introduced in the ligand set would tune the (i) amide electronic properties, (ii) effectiveness of protein–ligand interactions, and (iii) steric hindrance, all factors that could affect hydrolysis kinetic. To unravel this, we switched to a DFT (density functional theory) model (see Section 4), in order to characterize hydrolysis energy profiles (Figure 4). The approach consists of the construction of an all-QM (quantum mechanical) cluster model, starting from the CovDock poses obtained at the previous stage. The model comprises the catalytic triad and all the other residues (properly truncated) which interact with the docked ligands and/or limit the volume of the catalytic pocket. The proposed all-QM methodology is novel in the context of FAAH computational modeling and is an alternative to previously reported methodologies [19] falling in the QM-MM (quantum mechanics/molecular mechanics) framework [19,20,21,22,23,24,25]. In these works, the QM part, limited to the sole catalytic triad and to the polar head (plus a variable number of carbon atoms, depending on each case study) of the ligand, has been treated at a semiempirical level. The all-QM approach used in the present work explicitly includes the treatment of dispersion forces, allowing the quantification of the energetic contributions associated with both the covalent and non-covalent interactions at a higher accuracy level. This issue is crucial for the detection of eventual energy differences in the hydrolysis process resulting from ligands with very subtle structural differences. The calculated energy profiles depict only the first stage of hydrolysis, namely to the acyl enzyme intermediates, since it is reasonable to assume that the energy cost associated with enzyme de-acylation, assisted by a water molecule, would be identical for all of the ligands. The energy profiles have been calculated only for a selected subset of representative ligands (PEA, RePEA, **4**, **6**, **7**, **8**, MePEA1, MePEA2), selected to verify the effects on hydrolysis kinetics and thermodynamics deriving from (i) inversion of the amide bond, thus going from PEA-like to RePEA-like ligands, (ii) addition of heteroatoms in proximity to the amide group, and (iii) introduction of a methyl substituent in the polar head.

The lowest energy mechanism associated with FAAH acylation, as characterized by DFT calculations, is reported in Figure 4 and the results are compatible with previously proposed mechanisms [20,23]. The resting state (A) evolves through a concerted proton transfer, assisted by the catalytic triad, which ends with the deprotonation of Ser241 (B). The latter acts as a nucleophile attacking the amide carbonyl and forming the tetrahedral intermediate (C). A second concerted proton transfer (from Lys142 to Ser217) assists the leaving group protonation by Ser217 (D) and the acyl-enzyme is thus formed (E). Intermediate D is predicted to be transient, as expected, so for half of the tested ligands, it should not be formed at all along the catalytic cycle. This suggests that steps C→D and D→E can easily occur in a concerted manner, which also emerged from previous computational investigations [20]. The first step A→B represents an intermolecular proton transfer whose energy is independent from the nature of the ligand. This step is predicted to be facile, both kinetically and thermodynamically. Indeed, intermediate B is found to be rather stable, since the negatively charged Ser241 thiolate is strongly stabilized by interactions with the side chain of Ser217 and the backbone of Gly216. Interestingly, this result is novel with respect to previous theoretical investigations, which suggested A→B and B→C to be essentially concomitant [21,24]. The rate-determining step for PEA hydrolysis is the nucleophilic attack by Ser241, which also emerged from previous investigations on other ethanolamides, such as oleamide, although our calculated activation barrier is smaller (11.3 kcal/mol) than the one predicted for oleamide at different levels of theory (~18 kcal/mol) [22]. However, if considering the energetic span of the profile, calculated taking into account the energy separation between the lowest intermediate and the highest transition state, we obtained a value of 18.9 kcal/mol. Interestingly, this value is very close to the experimental activation barrier of 16 kcal/mol for the hydrolysis of oleamide, which is structurally related to PEA [26].

The calculated energy profiles suggest that two specific structural features should have a high impact on hydrolysis kinetics.

The first one is the inversion of the amide bond, going from PEA-like to RePEA-like ligands, which causes a doubling of the calculated catalysis energetic span (Figure 4, top, purple shades profiles). By reverting the amide bond, in fact, the oxyanion formed upon nucleophilic attack is not sufficiently stabilized by the oxyanion hole (Supplementary Figure S3A). This would translate into a more difficult hydrolysis process compared to the one of PEA.

A second structural feature that could hinder hydrolysis is the introduction of some steric bulk in the molecular structure, such as a methyl group, at the C-α of PEA. The calculated energy profile for MePEA1 lies, in fact, significantly higher in energy with respect to the one of PEA (Figure 4, top, light green energy profile). This result, which is in agreement with previous observations regarding anandamide vs. methanandamide behaviors [6], can be ascribed to the steric hindrance and electrostatic repulsion that are caused by introducing the methyl group (hydrophobic) in proximity to the oxyanion hole (hydrophilic) (Supplementary Figure S3B). This is supported by the fact that the shift of the methyl group in beta position, in MePEA2, is not predicted to alter hydrolysis kinetics with respect to PEA one (Figure 4, top, dark green energy profile), as a result of the absence of steric clashes with the protein matrix.

### 2.5. Ligand Binding to NAAA and Implications on Hydrolysis

To further investigate the behavior towards the hydrolysis of PEA and its analogues, we tested in silico their binding mode to human NAAA. According to the postulated mechanism, the N-terminal Cys126 participates in catalysis in zwitterionic form, as a Cys-S^−^/Cys-NH_3_^+^ ion pair, being both the nucleophile and proton source [27,28]. First, the thiolate attacks the carbonyl, forming the tetrahedral oxyanion intermediate, and then the proton is transferred from the α-amino group of Cys126 to the ligand, allowing the release of the leaving group. Although the tetrahedral intermediate is supposed to be elusive [29], an active role has been proposed for the backbone of Glu195 and the sidechain of Asn287 in stabilizing the oxyanion formed upon nucleophilic attack [30].

The poses obtained upon covalent docking of PEA and analogues are very similar: the acyl chain is enclosed in a very narrow hydrophobic channel, while the polar head in the calyx-shaped cavity is exposed to the solvent (Figure 2D). In all the top score poses of each ligand, we find the expected H-bond pattern involving the oxyanion hole (Glu195, Asn287) and the negatively charged oxygen atom of the tetrahedral intermediate. These observations suggest that all the tested ligands can be recognized and similarly bound by NAAA (see Supplementary Figure S4 for further details on ligand interactions and scores). Another frequent interaction involves Asp145 and the ligand polar head, in line with the proposal that this residue should assist enzyme turnover by shuttling protons to/from Cys126 [27,29].

**Figure 5:**
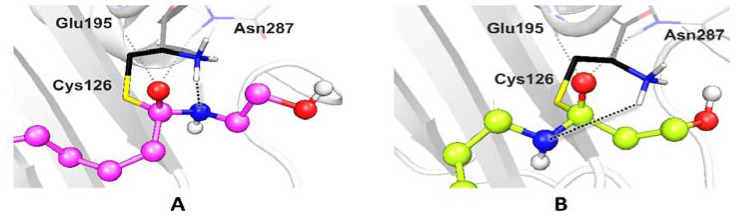
Comparison of the docking poses of PEA (**A**) and RePEA (**B**) into the NAAA pocket. In both cases, the oxyanion hole (Glu195 and Asn287) interacts with the negatively charged oxygen atom of the ligand. By reverting the amide bond, however, effective interaction between the NH_3_^+^ terminal of Cys126 and the leaving group of the ligand is hindered.

The simulated structure of PEA tetrahedral intermediate resembles the one modelled on the crystal structure of conjugated bile acid hydrolase (CBAH), another N-terminal cysteine hydrolase sharing with NAAA a highly conserved portion of the catalytic N-terminal region [31]. In PEA-like analogues, the amine of the ethanolamine head directly points toward Cys126-NH_3_^+^, a feature that should indicate a fast Cys126-to-ligand proton transfer (Figure 5A). If the amide bond is reverted, instead, as in RePEA-like analogues, the amine group of the ligand is shifted and located at a significant distance from to Cys126-NH_3_^+^ (Figure 5B). This would suggest that the Cys126-to-ligand proton transfer should be hindered unless we invoke important conformational rearrangements or alternative mechanisms for hydrolysis.

The computational investigation performed on the three targets suggests that the most strategic structural modifications to introduce in the PEA scaffold, in order to hinder hydrolysis, are reverting the amide bond and/or introducing steric bulkiness in the proximity of the amide. Particularly, among the RePEA-like compounds, RePEA is the best candidate in terms of both synthetic accessibility and metabolic stability to FAAH-mediated hydrolysis.

### 2.6. RePEA Is Stable When Exposed to FAAH-Containing Membranes

**Figure 6:**
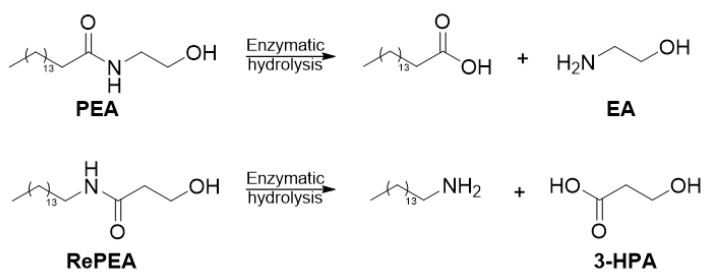
Enzymatic hydrolysis of PEA and RePEA affording ethanolamine (EA) and 3-hydroxypropionic acid (3-HPA), respectively.

**Figure 7:**
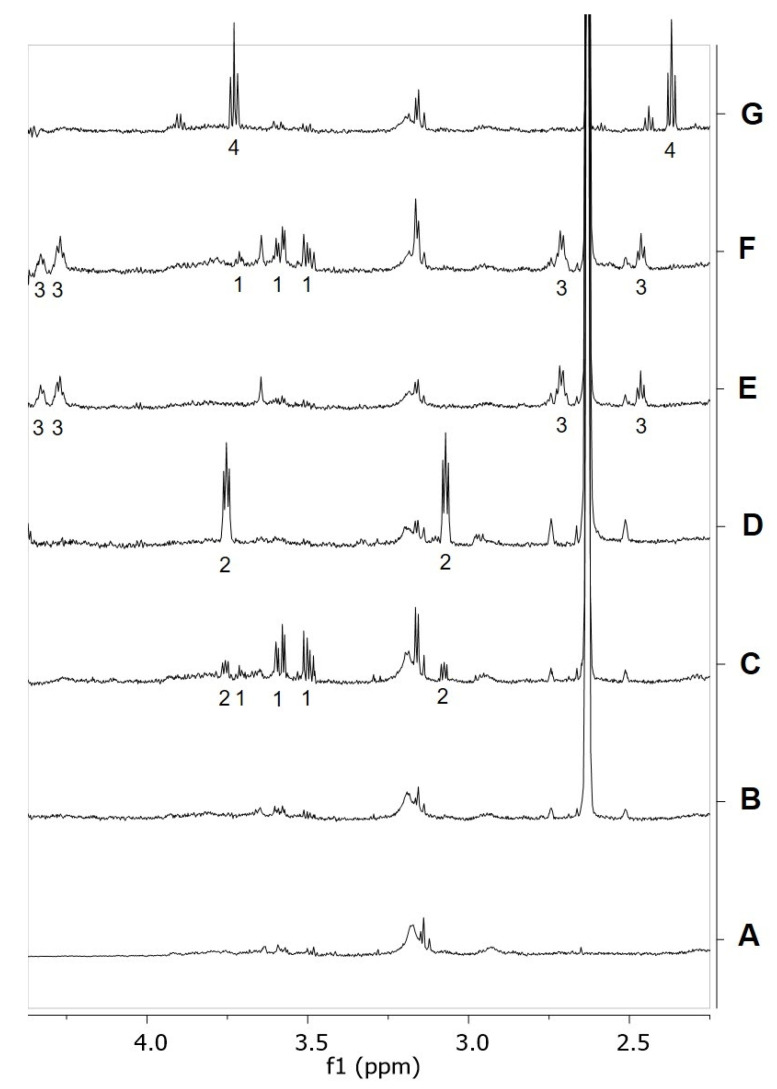
(**A**) ^1^H NMR spectrum of a membrane sample dissolved in deuterated PBS, pH 7.4, 37 °C. (**B**,**C**) ^1^H NMR spectra of the same sample after the addition of 1 mM PEA dissolved in d_6_-DMSO. Number of scans (NS) = 64; spectrum B recorded at t = 0 s after PEA addition, spectrum C recorded after 12 h; (**D**) ^1^H NMR spectrum of a membrane sample spiked with 0.1 mM ethanolamine (EA) dissolved in deuterated PBS, 10% d_6_-DMSO, pH 7.4, 37 °C; (**E**,**F**) ^1^H NMR spectra of the same sample after addition of 1 mM RePEA dissolved in d_6_-DMSO; spectrum E recorded at t = 0 s after RePEA addition, spectrum F recorded after 12 h; (**G**) ^1^H NMR spectrum of a membrane sample spiked with 0.1 mM 3-hydroxypropanoic acid (3-HPA) dissolved in deuterated PBS, 10% d_6_-DMSO, pH 7.4 37 °C. PEA NMR resonances are not visible in spectra B and C due to PEA interaction in membranes. 1: glycerol, 2: EA, 3: RePEA, 4: 3-HPA.

In order to validate DFT model predictions, PEA and RePEA hydrolysis, affording ethanolamine (EA) and 3-hydroxy-propionic acid (3-HPA), respectively (Figure 6), were monitored by ^1^H-NMR spectroscopy over 24 h (Figure 7 and Supplementary Figure S5A,B).

To membrane samples purified from frozen primary cortical cultures (cerebral cortices) from postnatal mice and containing FAAH as an integral membrane protein (a representative spectrum of membrane samples is depicted in Figure 7A), 1 mM of either PEA (Figure 7B) or RePEA (Figure 7E) was added and the mixtures were incubated in deuterated PBS, pH 7.4 with 10% d_6_-DMSO, at 37 °C. For each sample, a series of ^1^H-NMR spectra was recorded in 30 min intervals over 24 h (Supplementary Figure S5) to check the formation of the hydrolysis products. EA and 3-HPA NMR resonances are clearly highlighted in reference spectra (Figure 7D,G respectively) acquired on membrane samples spiked with the two compounds at a concentration of 0.1 mM. While EA resonances are also detectable in spectra acquired on membrane samples incubated with PEA (Figure 7C and Supplementary Figure S5A), 3-HPA signals did not appear after the addition of RePEA to membrane samples (Figure 7F and Supplementary Figure S5B). These findings definitely indicate that, under the experimental conditions described, PEA is partially converted to EA by FAAH, in contrast with RePEA, which appears to be resistant to FAAH-mediated hydrolysis.

Notably, in both cases the NMR signal of glycerol and other small metabolites appeared in the NMR spectra over time, suggesting their slow release from the membrane during kinetic monitoring.

### 2.7. PEA and RePEA Inhibits LPS-Induced Tumor Necrosis Factor-α (TNF-α) and Interleukin-6 (IL-6) Release in N9 Microglial Cells

To establish the bioactivity of PEA and its analogues, murine N9 microglial cells were used. The N9 microglia cells were developed by immortalizing primary microglia cells with the *v-myc* or *v-mil* oncogenes of the avian retrovirus MH2 [32] and are commonly used as an inflammatory model as they upregulate the pro-inflammatory genes, including inducible nitric oxide synthase (iNOS), cyclooxygenase-2, TNF-α and interleukin-1β (IL-1β) [33].

In order to select the concentration of compounds to be used, we performed preliminary experiments showing that PEA treatment (1–100 nM for 24 h) did not induce significant cellular cytotoxicity on murine microglia cells. In addition, the same concentration of PEA demonstrated a significant modulatory effect on rat microglia [34]. Therefore, we also examined the toxicity of 100 nM PEA analogues (RePEA, MePEA1, MePEA2).

**Figure 8:**
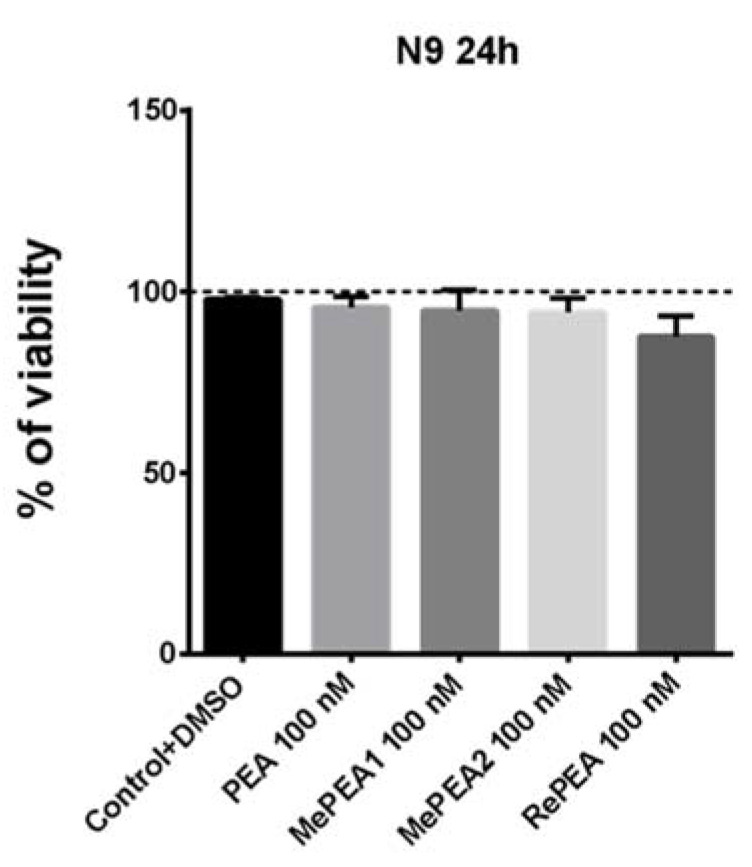
Cell viability in N9 murine microglial cells incubated for 1 h with 100 nM PEA and its analogues (MePEA1, MePEA2, RePEA). Then, medium was replaced with fresh IMDM supplemented with 5% FBS. Cell viability was quantified after 24 h. Data are presented as mean ± S.E.M. (*n* = 3 independent experiments).

Murine N9 microglia cells were incubated for 1 h with 100 nM of each compound and after 24 h cell viability was analyzed by MTT assays. Cells treated with vehicle (DMSO) were used as control. As is shown in Figure 8, none of the compounds had any effects on cell viability and they were not cytotoxic.

**Figure 9:**
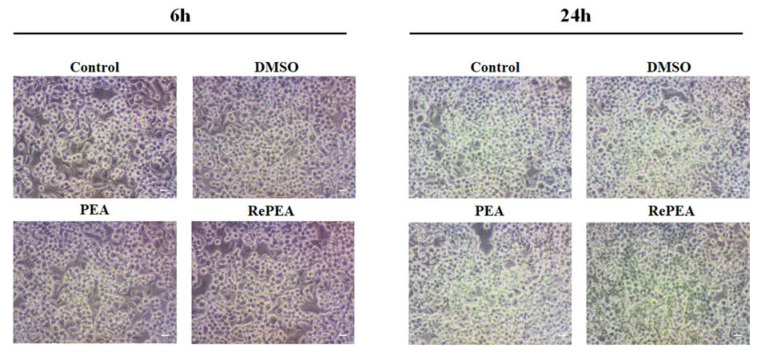
Cellular morphology of N9 cells incubated for 1 h with 100 nM PEA or RePEA. Then, medium was replaced with fresh IMDM supplemented with 5% FBS. After 6 or 24 h optical images were captured with an inverted Olympus CKX41 microscope. Representative images out of at least three separate experiments are shown. Scale bar: 10 µm.

To confirm MTT results, N9 cells were also analyzed by inverted microscopy (Figure 9 and Supplementary Figure S6) that revealed no changes in cell morphology between N9 cells treated with PEA or its analogues and cells not treated or treated with vehicle (DMSO).

**Figure 10:**
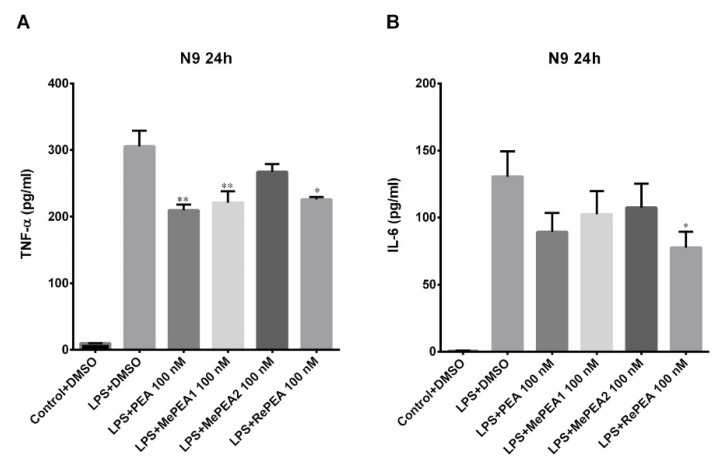
Release of TNF-α and of IL-6 at 24 h after LPS application. The mouse N9 microglial cells were stimulated with LPS at 10 ng/mL for 1 h; then, LPS-containing medium was removed and cells were incubated for 1 h with 100 nM PEA and its analogues (MePEA1, MePEA2, RePEA). Then, medium was replaced with fresh IMDM supplemented with 5% FBS. The amount of TNF-α (**A**) and of IL-6 (**B**) released into the culture medium was quantified after 24 h. Data are presented as mean ± S.E.M. (*n* = 3 independent experiments). Significant differences from LPS were determined using nonparametric one-way analysis of variance (ANOVA) with post hoc Dunnett’s multiple comparison tests at * *p* < 0.05; ** *p* < 0.01.

Microglia responses in inflammation have been extensively triggered by the use of lipopolysaccharide (LPS), a wall component of Gram-negative bacterial cells; in particular, LPS binds the CD14/TLR4/MD2 receptor complex on cell membranes and induces responses such as release of inflammatory mediators, e.g., TNF-α IL-6, and IL-1β [35,36]. Initially, to test the ability of PEA and its analogues to inhibit TNF-α release, N9 cells were stimulated with LPS for 1 h; cells were then treated with 100 nM of PEA or with its analogues (1 h) and TNF-α release was examined at 3 h, 6 h, and 24 h. No effect was observed at 3 and 6 h time points (data not shown). After 24 h, PEA, MePEA1, and RePEA inhibited TNF-α release, while MePEA2 appeared to be less effective (Figure 10A).

Afterwards, the release of IL-6 and IL-1β was examined at 24 h. As it is shown in Figure 10B only RePEA was able to counteract the release of IL-6 and this result is statistically significant in respect of LPS. Unfortunately, IL-1β release was not detectable and the effect of PEA and its analogues could not be assessed. This is not surprising as the release of IL-1β from mouse microglial cells is a very inefficient process and needs two different treatments (LPS and ATP) to be detectable [37].

### 2.8. PEA and RePEA Effects on Nuclear Factor Kappa-Light-Chain-Enhancer of Activated B Cells (NF-κB) Activation in Human PMA-THP-1 X-Blue™ Cells

Often mouse and human cells give rise to different results. As PEA analogues have been generated to be used on human beings, the experiments previously performed on mouse N9 microglial cells were repeated on PMA-THP-1 X-Blue™ human cells. Moreover, between the proposed compounds, only RePEA functioned at different times in N9 cells therefore the assays were performed using only PEA and RePEA.

At first, viability assays (MTT assay) and morphology analyses were performed at 24 h, the time at which TNF-α release has given the best results. The experiments show that PEA and RePEA were not cytotoxic and the morphology was not influenced by their presence (Figure 10B and Supplementary Figure S7).

**Figure 11:**
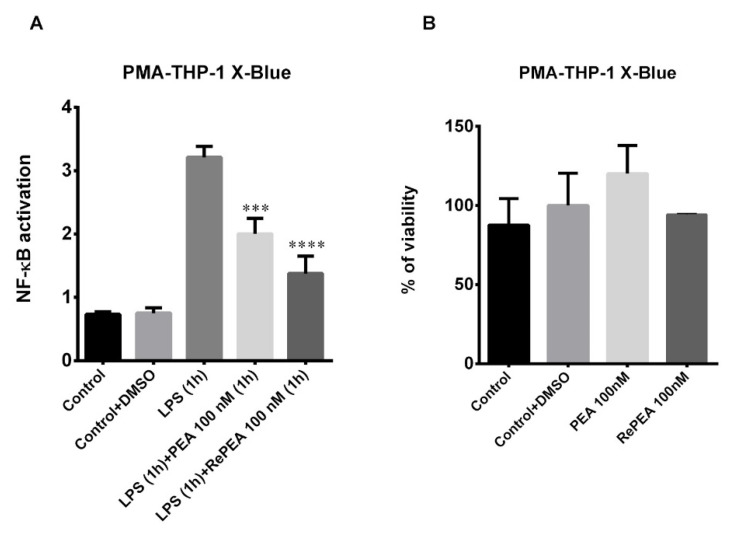
(**A**) Inhibition of NF-κB activation in LPS-stimulated PMA-THP-1 X-Blue™ cells by PEA and RePEA. PMA-THP-1 X-Blue™ cells were stimulated with LPS at 10 ng/mL for 1 h; then, the LPS-containing medium was removed and cells were incubated for 1 h with 100 nM PEA and RePEA. Then, the medium was replaced with fresh RPMI. The amount of SEAP (secreted embryonic alkaline phosphatase) released into the culture medium was quantified after 24 h as a measure of NF-κB activation. Data are presented as mean ± S.E.M. (*n* = 3 independent experiments), normalized on MTT data (the activity of SEAP, expressed as OD, was normalized on the MTT OD value of each corresponding well, as a measure of cell viability). Significant differences from LPS were determined using nonparametric one-way analysis of variance (ANOVA) with post hoc Dunnett’s multiple comparison tests at *** *p* < 0.001, **** *p* < 0.0001. (**B**). Cell viability in PMA-THP-1 X-Blue™ cells incubated for 1 h with 100 nM PEA and RePEA. Then, medium was replaced with fresh RPMI. Cell viability was quantified after 24 h. Data are presented as mean ± S.E.M. (*n* = 3 independent experiments).

Although structurally different, TNF-α, IL-1β, and toll-like receptors (TLR) use similar signal transduction mechanisms that include activation of IkB kinase (IKK) and NF-κB [38]. In order to obtain further insight into the role of the TLR4/NF-κB axis in the previously observed inhibition of pro-inflammatory cytokines, NF-κB activation triggered by TLR4 stimulation was investigated in PMA-THP-1 X-Blue™ cells. PMA-THP-1 X-Blue™ cells were used as a tool to investigate whether PEA and/or RePEA act involving TLR4 directly. Cells were treated as described in Materials and Methods. As shown in Figure 11A, at 24 h, both PEA and RePEA inhibited NF-κB activation triggered by TLR4 stimulation in PMA-THP-1 X-Blue™. In particular, RePEA decreased SEAP (secreted embryonic alkaline phosphatase) release by 50% while PEA decreased it by about 30%.

**Figure 12:**
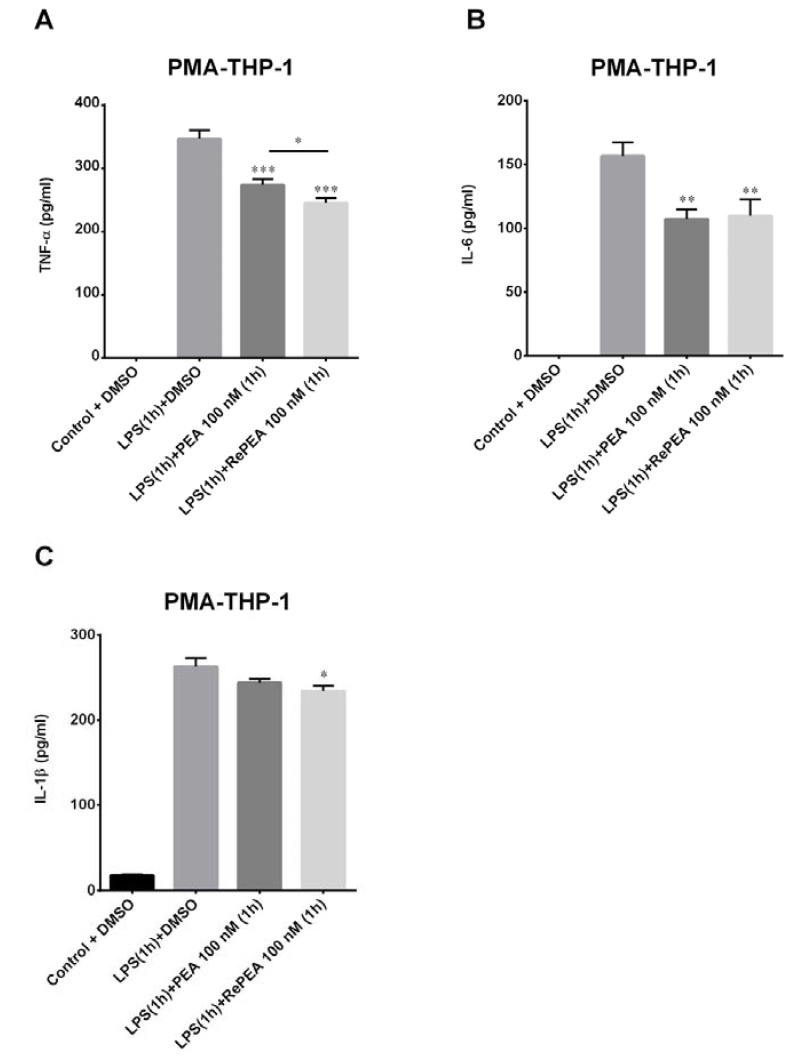
Release of TNF-α IL-6, and IL-1β at 24 h after LPS application. The human macrophages (PMA-THP-1) were stimulated with LPS at 10 ng/mL for 1 h; then, LPS-containing medium was removed and cells were incubated for 1 h with 100 nM PEA and its analogue, RePEA. Then, medium was replaced with fresh RPMI. The amount of TNF-α (**A**), IL-6 (**B**), and IL-1β (**C**) released into the culture medium was quantified after 24 h from LPS administration. Data are presented as mean ± S.E.M. (*n* = 3 independent experiments). Significant differences from LPS were determined using nonparametric one-way analysis of variance (ANOVA) with post hoc Dunnett’s multiple comparison tests at * *p* < 0.05; ** *p* < 0.01; *** *p* < 0.001. Difference between treatment with PEA and RePEA was determined using unpaired *t*-test * *p* < 0.05.

Furthermore, in order to test the ability of PEA and RePEA to counteract inflammation induced by LPS in PMA-THP-1 cells, macrophagic-release of TNF-α was evaluated. As shown in Figure 12, both PEA and RePEA decreased the amount of this cytokine triggered by LPS stimulation. Notably, RePEA inhibited TNF-α release compared to PEA in a very significant way. These results align with the hypothesis that RePEA is hydrolyzed slowly by FAAH. Finally, other cytokines associated with microglial activation were tested to confirm data obtained with TNF-α. In particular, IL-6 and IL-1β were analyzed. Released IL-6 and IL-1β cytokines were tested on PMA-THP-1 cells treated with LPS at 24 h. Cells were treated as described in Materials and Methods. As shown in Figure 12, IL-6 decreased significantly in respect of LPS both with PEA and RePEA treatments while IL-1β diminished only with RePEA treatment. These results confirmed previously obtained data and strengthened the potentiality of RePEA in the inhibition of pro-inflammatory cytokine release.

## 3. Discussion

PEA is an endogenous lipid mediator which is not stored in cells, but is rather synthesized on demand from membrane phospholipid precursors; its endogenous levels are regulated by enzymes responsible for its degradation to fatty acid and ethanolamine. In particular, two enzymes are known to play a central role in the inactivation of PEA by hydrolysis: FAAH, an intracellular serine hydrolase [39], and NAAA, a cysteine hydrolase localized in the lysosomes [40]. FAAH also plays an important role in the hepatic metabolism of PEA when it is exogenously administered. In fact, it has been recently proposed that whereas both FAAH and NAAA equally contribute to the catabolism of endogenous PEA, the FAAH-mediated degradation plays a predominant role in the metabolism of exogenously administered PEA [2]. The hydrolytic enzymes involved in PEA metabolism are expressed in the intestine and the liver [5] and the contribution of this pre-systemic metabolism strongly affects PEA bioavailability. This metabolic instability affects the pharmacokinetic properties of PEA which remain the main issue of therapeutic use in humans. In fact, in spite of the amelioration in the dissolution rate and absorption elicited by new oral formulations of PEA (such as micronized- and ultramicronized-PEA) [41], the metabolic inactivation is responsible for the short-lasting effects of the compound. A significant number of clinical trials suggest that systemic administration of PEA exerts anti-inflammatory, immunomodulatory, and neuroprotective effects, but it is especially evaluated for chronic pain management in humans [42,43,44,45]. Importantly, clinical trials prove that exogenous administration of PEA is well tolerated; in fact, the lack of side effects is a common finding in most clinical studies, as reported by a recent review carrying out a pooled meta-analysis based on data available from clinical trials about PEA employment in pain-suffering patients [46]. The efficacy and the tolerability of PEA explain why since 2008 it has been marketed in different countries as a nutraceutical food supplement.

Strategies aimed to ameliorate the in vivo use of PEA are required. One strategy to decrease PEA degradation could be the development of FAAH inhibitors; however, these compounds should increase the endogenous levels of other substrates of FAAH. On the other hand, an alternative strategy should be the development of PEA analogues, more stable to the enzymatic inactivation.

Different molecular mechanisms have been proposed so far to explain the biological effects of PEA. PEA can act through the so-called “entourage effect”, increasing the level of other endocannabinoids which in turn activate cannabinoid receptors, or it can act via an allosteric modulation of TRPV1 receptors potentiating its activation by direct ligands [47]. Furthermore, it has been proposed that PEA can activate GPR55 receptor [48] even if this hypothesis awaits further evidence. Until now, the only direct target of PEA is PPAR-α receptor [1], which is considered the mediator of PEA anti-inflammatory effects. For this reason, we assessed whether the PEA analogues retain the ability to bind this pharmacological target of PEA.

Based on these issues, we designed a small library of PEA analogues, still able to maintain PPAR-α affinity and possessing longer life than their natural counterpart, being more stable to the hydrolytic action of FAAH. For comparison, we included in the analysis of the newly developed analogues PEA itself and two commercially available PEA analogues, i.e., MePEA1 and MePEA2.

Computational results suggest that MePEA1 and RePEA could be the ideal candidate to be hydrolyzed more slowly by FAAH, compared to PEA. As a matter of fact, methanandamide (Meth-AEA), a synthetic analogue of AEA, has a high resistance to enzymatic hydrolysis [6]. The same modification in PEA, yielding MePEA1, is expected to provide similar metabolic stability. Furthermore, it was reported that although RePEA cannot significantly inhibit FAAH [49], it can efficiently inhibit NAAA. This is probably related to the higher affinity of PEA analogues for NAAA [50], while anandamide analogues are preferred substrates for FAAH [51]. These data tally with computational results, suggesting that the DFT model was actually able to predict which compounds would be more resistant to hydrolysis. The major stability of RePEA was experimentally confirmed by its resistance when exposed to cell membranes. While PEA was prone to be partially hydrolyzed in 24 h, RePEA was persistent in the assay, confirming the robustness of the DFT model we proposed.

Molecular docking of PEA and its analogues to the binding domain of PPAR-α revealed a very similar binding mode, confirming that the modifications introduced to hinder hydrolysis of the amidic bond do not interfere with the affinity to the receptor. Although this result suggests that the analogues share with PEA the pharmacodynamic property of PPAR-α binding, research on the mechanism of action of these compounds is beyond the main aim of our work. Since many effects of PEA do not involve this receptor and various indirect mechanisms of action have been reported, we aimed to evaluate whether the compounds retained the ability of PEA to counteract inflammatory response in a widely employed cellular setting. All the computational data revealed RePEA as the best candidate in terms of both synthetic accessibility and metabolic stability to FAAH-mediated hydrolysis. Since our results focused on the stability to hydrolysis catalyzed by FAAH, we cannot foresee the general in vivo metabolic stability of RePEA. Only further characterization aimed to evaluate the compound stability in plasma as well as the determination of metabolites produced in liver homogenate or in hepatic cell lines will allow us to propose RePEA as an in vivo metabolically stable compound. Thus, RePEA was synthesized and submitted to the biological assays in order to determine whether it was able to retain the anti-inflammatory properties of PEA.

It is well known that microglial cells, the phagocytic resident macrophages in the brain, play a key role in regulating cerebral inflammatory reactions [52]. In normal conditions, microglial cells are in “resting state”. However, several agents can activate microglia to produce iNOS and pro-inflammatory factors which can induce neuron death [53]. In the present study, we used LPS to activate mouse N9 microglia cells and human macrophages and we evaluated the secreted TNF-α as the main pro-inflammatory marker. Other released pro-inflammatory cytokines were also evaluated, e.g., IL-6 and IL-1β. IL-6 is a cytokine with a dual effect; at some levels, it acts as a defense mechanism but, in chronic inflammation, it is rather proinflammatory. IL-6 has stimulatory effects on T- and B-cells and, in addition, in combination with its soluble receptor sIL-6Rα, tunes the transition from acute to chronic inflammation [54]. IL-1β is a potent pro-inflammatory cytokine that is crucial for host-defense responses to infection and injury [54,55]; it is involved in chronic inflammation such as rheumatoid arthritis, neuropathic pain, inflammatory bowel disease, osteoarthritis, vascular disease, multiple sclerosis, and Alzheimer’s disease [56]. LPS is a constituent of the outer membrane of Gram-negative bacteria and has been widely used to induce experimental inflammatory reactions [53,57]. This agent is powerful enough to activate microglia into M1 state [58]. The mouse N9 microglial cell line used in this study, like primary microglia, can be polarized into M1 or M2 state and secrete the markers of microglial M1 and M2 states, such as iNOS, TNF-α, IL-1β and Arg-1, in the presence of a stimulus [59,60]. Microglia activation connected to pro-inflammatory responses has been considered detrimental in particular for neurons, and drugs able to stop microglia activation have been proposed for the treatment of a variety of diseases [61,62,63,64]. In our experiments, PEA, MePEA1, and RePEA inhibited TNF-α release after 24 h, whereas MePEA2 seemed to have few effects. Similar results were obtained measuring the level of another pro-inflammatory cytokine, IL-6. RePEA decreased IL-6, while PEA at the same concentration could not induce any decrease. These data suggest that RePEA still retains the ability of PEA to counteract LPS-induced inflammatory responses. More importantly, the evaluation of the same effect on human macrophages highlights a higher potency of RePEA in controlling inflammation than PEA.

Microglia and macrophages express a wide range of receptors, including TLRs, a subfamily of pattern-recognition receptors that recognize invading pathogens and endogenous harmful stimuli, to induce innate and adaptive immune responses [65]. Among TLRs, TLR4 is the major LPS receptor [66,67]. When expressed on the cellular membrane, TLR4 exists as a complex with the co-receptor myeloid differentiation protein-2 (MD-2), which is essential for LPS recognition by the TLR4–MD-2 complex [68,69]. Binding of LPS causes the TLR4–MD-2 complex dimerization [70], which results in the activation of downstream mediators, including the nuclear transcription factor NF-κB, which increases the production of pro-inflammatory molecules, such as cytokines (e.g., TNF-α, IL-1β, and IL-6), chemokines, enzymes, and reactive oxygen and nitrogen species [71]. NF-κB is a well-known master regulator of inflammation. In recent years, it has become clear that there are at least two separate pathways for NF-κB activation, the “canonical” and the “non-canonical” pathway. TLR4, TNF-α receptor, and IL-1β receptor are known to stimulate NF-κB through the “canonical” pathway, as activation of these receptors leads to phosphorylation of IKK [72] and this process is mediated by the adapter protein MyD88 [73]. In the “non canonical” pathway, NF-κB-inducing kinase (NIK) activates IKKα that phosphorylates p100, which is converted into p52/RelB heterodimers [72]. So, NF-κB dimers, released from the IκB complex, translocate to the nucleus and bind to specific DNA sequences in the promoter of many genes [74,75]. Preclinical studies show the therapeutic effect of synthetic small molecules acting as TLR4 antagonists, both in vitro and in vivo, and confirm its central role in the regulation of inflammation [76]. Both PEA and even more RePEA inhibit NF-κB activation triggered by TLR4 stimulation. Moreover, no changes in cell morphology or viability were observed between N9 cells treated with PEA or its analogues, suggesting that none of the compounds are toxic.

In this work we proposed the design of a small library of PEA analogues and we developed a novel QM model that was able to predict the properties of the designed compounds and some commercial ones as far as FAAH-mediated hydrolysis is concerned. In conclusion, although the specific mechanism of action and the evaluation of in vivo metabolic stability awaits further investigation, RePEA represents a good candidate for pre-clinical studies in order to develop a compound with the same well-known therapeutic properties of PEA but with a better pharmacokinetic profile.

## 4. Materials and Methods

Palmitoylethanolamide (PEA, CAS N° 544-31-0), R-palmitoyl-(1-methyl) ethanolamide (MePEA1, CAS N° 142128-47-0), and R-palmitoyl-(2-methyl) ethanolamide (MePEA2, CAS N° 179951-56-5) were purchased from Cayman Chemical (Ann Arbor, MI, USA) with a declared purity ≥98%. 3-Hydroxy-*N*-pentadecylpropanamide (RePEA) was synthesized with a slight modification to methods previously reported in literature [49].

### 4.1. Chemical Procedures

Anhydrous solvents over molecular sieves were purchased from Acros Organics^®^ (Thermo Fisher Scientific, Geel, Belgium) with a content of water ≤50 ppm. Thin-layer chromatography (TLC) was performed on Silica Gel 60 F_254_ plates (Merck, Darmstadt, Germany) and visualized using appropriate developing solutions. Automated flash chromatography was performed on a Biotage^®^ Isolera™ (Biotage, Uppsala, Sweden). Prime system. NMR experiments were recorded on a Bruker Avance III 600 MHz equipped with cryo-probe instrument at 298 K. Chemical shifts (δ) are reported in ppm downfield from the residual solvent peak, whereas coupling constants (*J*) are stated in Hz. The ^1^H and ^13^C-NMR resonances of compounds were assigned by means of COSY and HSQC experiments. NMR data processing was performed with MestReNova v14.1.2 software (Mestrelab Research, Santiago de Compostela, Spain).

#### 4.1.1. Synthesis of 3-Hydroxy-N-pentadecylpropanamide (RePEA)

A solution of pentadecylamine (100 mg, 0.44 mmol) in anhydrous dichloromethane (1 mL) kept at 0 °C was treated with β-propiolactone (83 μL, 1.32 mmol). The mixture was then allowed to return to room temperature and stirred overnight under argon atmosphere. Then, the reaction was quenched with MeOH and concentrated under reduced pressure and the crude was purified by automated flash chromatography (Hex:AcOEt gradient elution) obtaining pure compound RePEA (60 mg, 48% yield purity ≥95%). TLC (ethyl acetate) Rf = 0.25; ^1^H NMR (600 MHz, CDCl_3_) δ 6.02 (bs, 1H, NH), 3.92–3.87 (m, 2H, H_3_), 3.27 (dd, = 6.8, 5.6 Hz, 2H, H_4_), 2.48 (t, *J_2,3_* = 4.8 Hz, 2H, H_2_), 2.38 (bs, 1H, OH), 1.51 (q, *J* = 6.8 Hz, 2H, H_5_), 1.35–1.27 (m, 8H, CH_2_), 1.25 (s, 16H, CH_2_), 0.88 (t, *J* = 7.0 Hz, 3H, H_18_). ^13^C NMR (150 MHz, CDCl_3_) δ 173.35 (CO), 59.04 (C_3_), 40.15 (C_4_), 37.57 (C_2_), 32.07, 29.83, 29.74, 29.67, 29.51 (C_5_), 29.40, 27.05, 22.84, 14.27 (C_18_).

#### 4.1.2. Sample Preparation

PEA, MePEA1, MePEA2, and RePEA were dissolved in dimethyl sulfoxide (DMSO) at a concentration of 67 mM, and then serially diluted in culture medium immediately prior to experiments. The final concentration of DMSO was less than 0.01% in the experiments. Lipopolysaccharides (LPS; Escherichia coli O55:B5) were obtained from ENZO Life Sciences (New York, NY, USA).

### 4.2. Computational Methods

#### 4.2.1. Molecular Docking

Molecular recognition simulations were performed using the docking tools Glide [77,78,79,80] and CovDock [81], as available in Maestro 12.1 (Schrodinger Inc., LLC, New York, NY, USA). The X-ray crystal structures corresponding to the different PDB IDs of the investigated proteins (1I7G for PPAR-α, 6DXX for NAAA and 3K84 for FAAH) were edited for missing hydrogens and for assigning proper bond orders. Water molecules and co-crystallized ligands were removed, while protonation states of side chains were assigned running PROPKA, setting pH = 7. The H-bonds were then optimized using sample orientations. Finally, a restrained minimization of proteins was carried out using the OPLS3 force field, [82,83] until the RMSD between the starting structure and the minimized one reached 0.3 Å. PEA and its analogues were geometrically refined with the “Ligprep” module using OPLS3 force field. The (*R*)-stereochemistry for MePEA1 and MePEA2 was retained, for consistency with experiments. Receptor grids were all calculated with the OPLS3 force field as well. The receptor grid for PPAR-α was generated by selecting the crystal structure co-ligand AZ 242 ((2S)-2-ethoxy-3-[4-(2-{4-[(methylsulphonyl) oxy]phenyl}ethoxy)phenyl]propanoic acid) as centroid. The grid box for FAAH and NAAA, instead, were built selecting active site amino acids Gly240, Phe192, Ala490 and Cys126, Asn287, Phe174, Trp181, respectively. For PPAR-α, flexible ligand docking using the extra precision (XP) Glide module was performed [80], with no constraints on receptor–ligand(s) interactions. The docking performance was validated by re-docking the alpha-ketoheterocycle co-ligand ((9Z)-1-(5-pyridin-2-yl-1,3,4-oxadiazol-2-yl) octadec-9-en-1-one) and by comparing the lowest energy pose with the co-ligand position and conformation found in the crystal structure. Sidechains of key residues Tyr464, Tyr314, His440 and Ser280 were set as freely rotable during calculations. For FAAH and NAAA, instead, covalent docking with the CovDock workflow was carried out [81]. Ser241 (for FAAH) and Cys126 (for NAAA) were set as reactive residues and, in both cases, the reaction type was defined as a nucleophilic addition to a double bond. FAAH was chosen as a reference system to validate the CovDock procedure for this kind of ligand and chemical reaction.

#### 4.2.2. Density Functional Theory Calculations

Quantum mechanical (QM) calculations were carried out in the density functional theory (DFT) framework, cutting off a cluster model from the FAAH crystal structure (PDB ID 3K84). The use of an all-QM cluster approach, instead of the alternative and more computationally costly hybrid QM-MM one (adopted for previous investigations on this theme) allowed us to treat at a more accurate DFT level a large portion of the protein active site, going beyond the sole catalytic triad.

The docking poses obtained with ligands covalent docking to FAAH were taken as the starting points to design the DFT model. In particular, we performed a cross-analysis of protein–ligand interactions found in the best poses of PEA and all the 10 analogues. In this way, we were able to identify crucial residues interacting with both the polar head and hydrophobic tail of our ligands. Since the structural variability among the ligands is introduced only in the polar head, the ligands’ length was reduced by truncating their hydrophobic chain, so that they are overall characterized by a 11-atom-long chain (comprising both carbons and heteroatoms). The residues included in the DFT model are 16: Ser241, Lys142, Ser217, Ser218, Ser190, Met191, Phe192, Glu240, Gly239, Ile238, Gly235, Thr236, Leu278, Cys269, Val270, Try271 (Supplementary Figure S8). The last four residues do not interact with the ligands but have been introduced in order to sterically define the volume of the pocket accommodating their polar head (vide infra). Geometry optimizations were carried out at the BP86/DZP level [84,85,86], using the TURBOMOLE 2.1 suite [87]. The resolution-of-identity (RI) approximation was used to speed up calculations [88]. The Grimme’s D3 corrections were added [89], in order to accurately account for the significant amount of dispersive protein–ligand interactions found in this system. Following a well-established approach, alpha carbons were kept frozen at the X-ray structure position. This constrained optimization is necessary to avoid unrealistic movements of amino acids within the active site. Full vibrational analysis was carried out to characterize the nature of transition states, searching for the imaginary frequency associated with the reaction coordinate of interest. Solvent effect was implicitly treated according to the COSMO approach, [90,91] by simulating a continuum dielectric with ε = 40 (which is a good compromise for a system that is half water accessible and half hydrophobic). The hydrolysis starting points were modeled as van der Waals adducts, obtained by dissociating the Ser241-ligand complexes obtained with CovDock, and by restoring the reactants functional groups.

### 4.3. FAAH Assay

Membranes for FAAH assay were prepared as described by Jonsson et al. with minor modifications [92]. Briefly, frozen primary cortical cultures (cerebral cortices) stored at −80 °C from postnatal mice were thawed, homogenized on ice in cold PBS (PH 7.4) using an insulin syringe and centrifuged at 13,000 rpm for 30 min. The cell pellets were then washed twice with PBS and centrifuged at 13,000 rpm for 30 min. The pellets were suspended in cold PBS on ice and sonicated. Protein concentration was measured and samples were stored at −80 °C until use.

The hydrolysis of PEA and RePEA was followed by NMR spectroscopy, using a Bruker Avance III NMR spectrometer equipped with a QCI cryogenic probe.

For each NMR sample, 50 µL of membrane (167 µg of total protein) was resuspended in 148 µL of deuterated PBS 10 mM, pH 7.4, and transferred into a 3 mm NMR tube. A first proton was acquired at 37 °C using the pulse sequence noesygppr1d and 64 scans, a relaxation delay of 2 s, an acquisition time of 2 s and a receiver gain of 45.2. Then, the potential FAAH substrates PEA or RePEA were added immediately starting the reaction monitoring by applying the same acquisition parameters. Due to their very low water solubility, PEA and RePEA were added after dissolution in d6-DMSO, reaching a final substrate concentration of 1 mM and 10% of d6-DMSO. Spectra were acquired every 30 min over an interval of 24 h. They were processed using MestreNova software version 14.1.2-25024 (Mestrelab Research, Santiago de Compostela, Spain) by applying a line broadening of 0.3 Hz. Spectra were referenced to DMSO residual signal.

### 4.4. Cell Cultures

#### 4.4.1. Murine N9 Microglial Cells

The murine microglial N9 cells were cultured in Iscove Modified Dulbecco’s Medium (IMDM, Sigma-Aldrich, St. Louis, MO, USA) supplemented with 5% heat-inactivated fetal bovine serum (FBS), 100 IU/mL penicillin, 100 U/mL streptomycin, 2 mM L-glutamine (all Euroclone, Pero, Italy), and Mycozap^™^ prophylactic (Lonza, Walkersville, MD, USA) under standard cell culture conditions (37 °C, 5% CO_2_).

#### 4.4.2. THP-1 and THP-1 X-Blue™ Cells

THP-1 and THP-1 X-Blue™ cells were maintained in RPMI 1640 Medium without L-glutamine with phenol red (Euroclone, Pero, Italy), supplemented with 10% heat-inactivated fetal bovine serum (FBS) (Euroclone, Pero, Italy), 2 mM L-glutamine (Euroclone, Pero, Italy), and 100 U/mL penicillin/streptomicin (Euroclone, Pero, Italy). Before treatments, cells were seeded into a 96-well plate and differentiated into macrophages by 72 h incubation with 100 ng/mL phorbol 12-myristate 13-acetate (PMA, Enzo Life Sciences, New York, NY, USA) followed by 24 h incubation in RPMI medium.

### 4.5. Cell Viability Assay

N9 cells were seeded at the concentration of 3 × 10^4^ cells/well into a 96-well plate and then the day after were incubated for 1 h with 100 nM PEA and its analogues (MePEA1, MePEA2, RePEA). Then, the medium was replaced with fresh IMDM supplemented with 5% FBS. After 24 h, cells were washed and incubated with fresh medium containing 3-(4,5-dimethylthiazol-2-yl)-2,5-diphenyltetrazolium bromide (MTT) (0.5 mg/mL; Sigma-Aldrich, St. Louis, MO, USA) at 37 °C for 4 h. After, formazan crystals were dissolved in acidic isopropanol. The optical density was evaluated by spectrophotometric measurement of absorbance. All the experiments were done in triplicate and repeated for three independent measurements.

### 4.6. Morphological Analysis

N9 and THP-1 cells were seeded at the concentration of 9 × 10^4^ cells/well and 8 × 10^4^ cells/per well respectively into a 96-well plate. THP-1 cells were differentiated as described above. Cellular morphology of N9 and PMA-THP-1 cells was evaluated using inverted Olympus CKX41 microscope (Olympus Instruments, Tokyo, Japan), equipped with a Digital C-Mount Camera TP 5100. Cells were incubated for 1 h with 100 nM PEA and its analogues (MePEA1, MePEA2, RePEA). Then, medium was replaced with fresh IMDM supplemented with 5% FBS (N9 cells) or RPMI (PMA-THP-1 X-Blue™ cells). After 6 or 24 h optical images were captured with an inverted microscope (Olympus CKX41, Olympus Instruments, Tokyo, Japan).

### 4.7. LPS Treatment with and without PEA and Its Analogues

N9, PMA-THP-1 X-Blue™, and PMA-THP-1 cells were stimulated with LPS at 10 ng/mL for 1 h, then the medium that contained LPS was removed and cells were incubated for 1 h with 100 nM PEA and its analogues (MePEA1, MePEA2, RePEA). Then, the medium was replaced with fresh IMDM supplemented with 5% FBS (N9 cells) or RPMI (PMA-THP-1 X-Blue™ cells).

### 4.8. Pro-Inflammatory Cytokine Release

N9 cells were seeded at the concentration of 9 × 10^4^ cells/well into a 12-well plate and then the day after were treated as described above. Levels of TNF-α released into the culture medium were quantified after 3, 6, and 24 h by using the corresponding quantification enzyme-linked immunosorbent assay (ELISA) kits (88-7324, Thermo Fisher Scientific, Monza, Italy) according to the manufacturer’s instructions. Levels of IL-6 and IL-1β released into the culture medium were quantified after 24 h by using the corresponding quantification enzyme-linked immunosorbent assay (ELISA) kits (DY406, DY401, R&D systems, Minneapolis, MN, USA). PMA-THP-1 cells were seeded at a concentration of 8 × 10^4^ cells/per well into a 96-well plate and differentiated as described above. Cells were then treated with 100 nM PEA or RePEA as indicated above. Levels of TNF-α, IL-6, and IL-1β released into the culture medium were quantified after 24 h by using the corresponding quantification enzyme-linked immunosorbent assay (ELISA) kits (DY210, DY206, DY201, R&D systems, Minneapolis, MN, USA) according to the manufacturer’s instructions. The data were expressed as pg/mL following interpolation on the basis of a standard curve. The experiment was done in triplicate and repeated for three independent measurements.

### 4.9. SEAP Assay

THP-1 X-Blue™ NF-κB cells were specifically designed for monitoring the NF-κB signal transduction pathway in a physiologically relevant cell line. THP-1-Blue™ is derived from the human THP-1 monocyte cell line by stable integration of an NF-κB-inducible secreted embryonic alkaline phosphatase (SEAP) reporter construct. THP-1-Blue™ NF-κB cells are highly responsive to PRR agonists that trigger the NF-κB pathway. THP-1 X-Blue™ NF-κB cells express a SEAP reporter gene driven by an IFN-β minimal promoter fused to five copies of the NF-κB consensus transcriptional response element and three copies of the c-Rel binding site.

A total of 8 × 10^4^ cells/per well were seeded into a 96-well plate and differentiated as described above. Cells were then treated with 100 nM PEA or RePEA as indicated above. As a result, PMA-THP-1 X-Blue™ NF-κB cells allow the monitoring of NF-κB activation by determining the activity of SEAP. Levels of SEAP in the supernatant have been easily determined after 24 h with Quanti-Blue™ solution according to manufacturer instructions (InvivoGen, San Diego, CA, USA). In addition, the cell density of each well was analyzed by MTT assay as described above. Activity of SEAP, expressed as OD, was normalized on the MTT OD value of each corresponding well, as a measure of cell viability.

### 4.10. Statistical Analysis

Results are expressed as the mean ± SEM. All data were analyzed using the GraphPad Prism software (version 6.0) (San Diego, CA, USA). Differences between treatment groups were analyzed using one-way analysis of variance (ANOVA), followed by post hoc Dunnett’s test or *t*-test. Difference between treatment with PEA and RePEA was determined using unpaired *t*-test. A p-value of less than 0.05 was considered statistically significant.

## Supplementary Materials

Supplementary Materials can be found at https://www.mdpi.com/1422-0067/21/23/9074/s1.

## Abbreviations

ABP	Acyl-chain Binding Pocket
AF-2	Activation function-2
DFT	Density Functional Theory
EA	Ethanolamine
FAAH	Fatty Acid Amide Hydrolase
HPA	Hydroxy-propionic acid
IL-1β	Interleukin-1β
IL-6	Interleukin-6
iNOS	inducible Nitric Oxide Synthase
LPS	Lipopolysaccharide
MAGL	Monoacylglycerol lipase
MD-2	Myeloid differentiation protein-2
MePEA	Methan-PEA
NAAA	*N*-acylethanolamine acid amidase
NF-κB	Nuclear Factor kappa-light-chain-enhancer of activated B cells
PEA	PalmitoylEthanolAmide
PPAR-α	Peroxisome Proliferator-Activated Receptor alpha
QM	Quantum Mechanical
QM-MM	Quantum Mechanics/Molecular Mechanics
RePEA	Retro-PEA
SEAP	Secreted embryonic alkaline phosphatase
SIFs	Structural Interaction Fingerprints
TLR	Toll Like Receptor
TNF-α	Tumor necrosis factor alpha
