# Supplementary material for: Synthesis, Molecular Modeling and Biological Evaluation of Metabolically Stable Analogues of the Endogenous Fatty Acid Amide Palmitoylethanolamide
Source: Int J Mol Sci. 2020 Nov 28;21(23):9074. doi: 10.3390/ijms21239074 (PMC7730713; doi:10.3390/ijms21239074)
Supplement: Supplementary file 1 [file ijms-21-09074-s001.pdf]

Supplementary Materials

Synthesis, Molecular Modeling and Biological Evaluation of Metabolically Stable Analogues of the Endogenous Fatty Acid Amide Palmitoylethanolamide

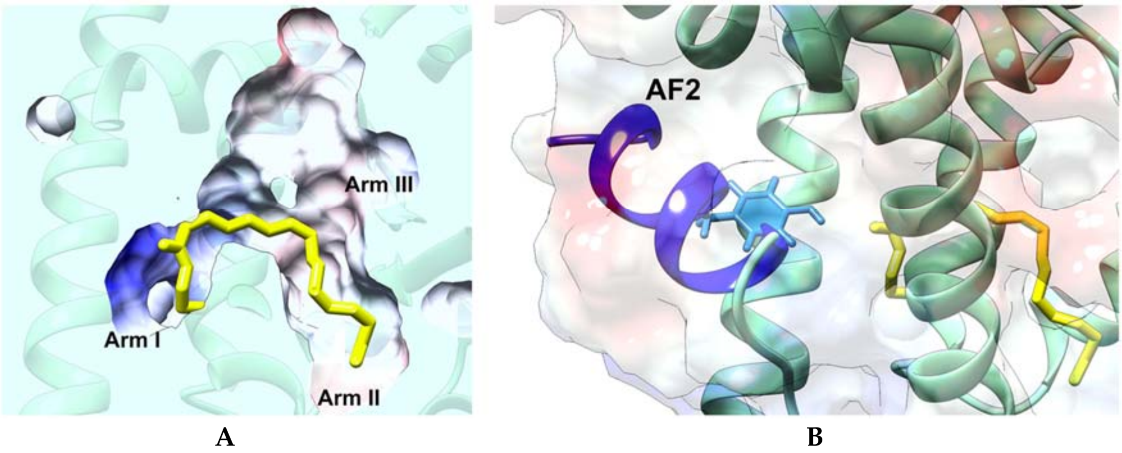

Figure S1. (**A**) **PEA** (in yellow) docked into the PPARα ligand binding domain. The different electrostatic nature of the catalytic pocket arms (arm I, arm II and arm III) are highlighted. (**B**) PEA, docked into the PPARα pocket, interacting with helix AF2. The latter is colored in blue. Tyr464 is shown as light blue sticks.

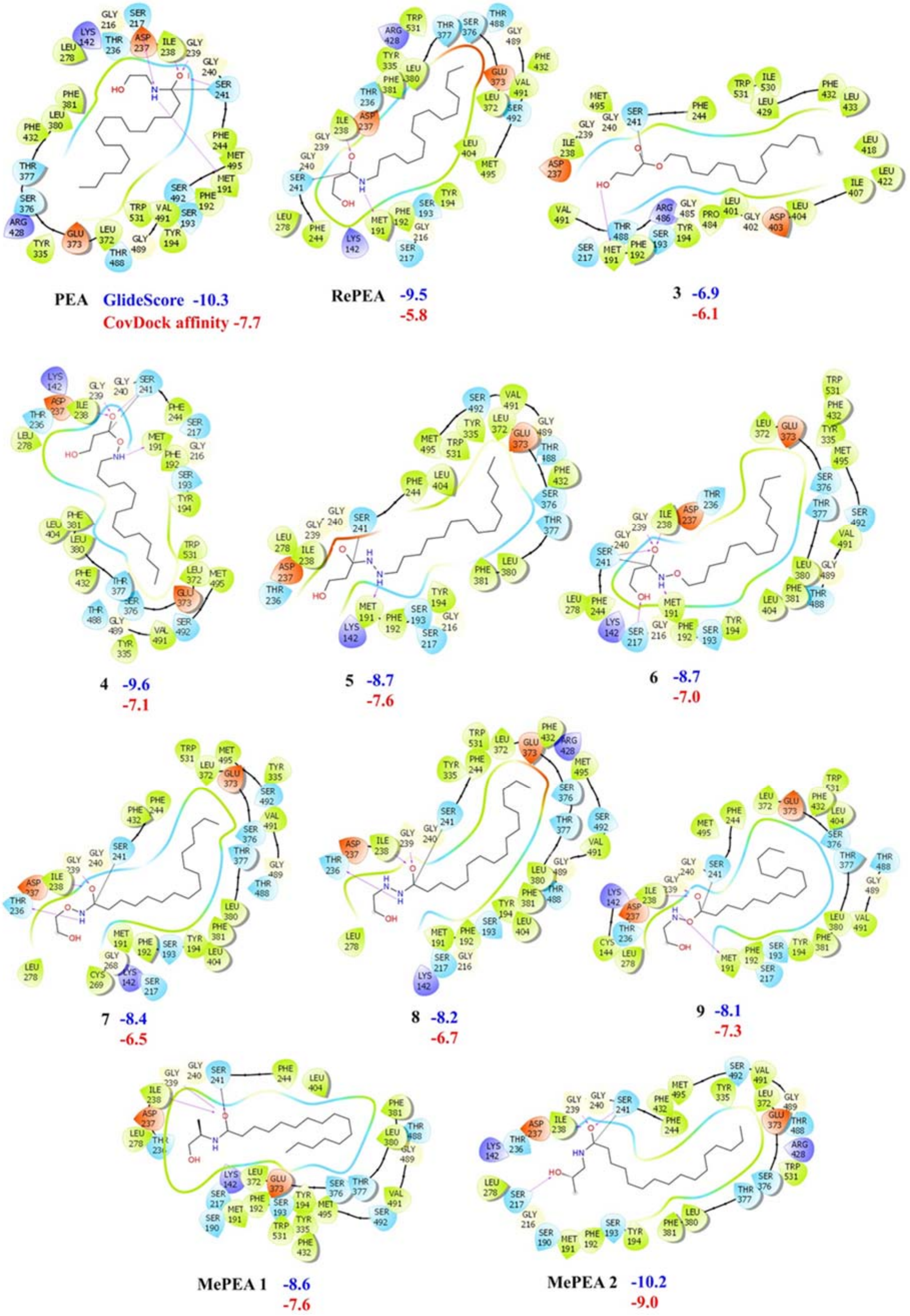

Figure S2. 2D ligand interaction diagrams of **PEA** and analogues with FAAH. GlideScores (kcal/mol, blue values) and CovDock affinities (kcal/mol, red values) are also reported.

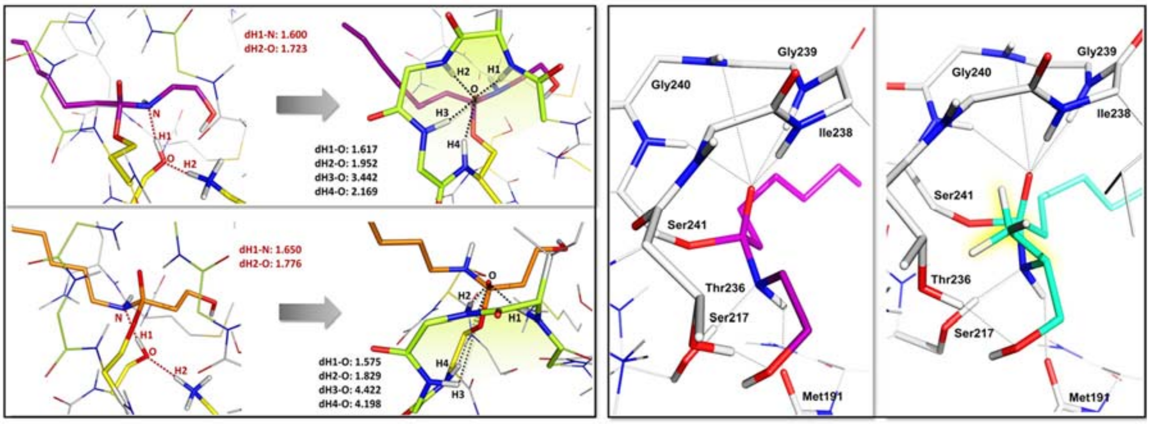

A **B**

Figure S3. (**A**) DFT-optimized structure of **PEA** (in purple) and **RePEA** (in orange) tetrahedral intermediates. The interaction between the ligands and the oxyanion hole (represented in light green) is highlighted. Main geometrical parameters are reported in Å. (**B**) DFT-optimized structure of **PEA** (in purple) and **MePEA 1** (in green) tetrahedral intermediates. The methyl group, creating steric hindrance within the active site pocket, is highlighted in yellow.

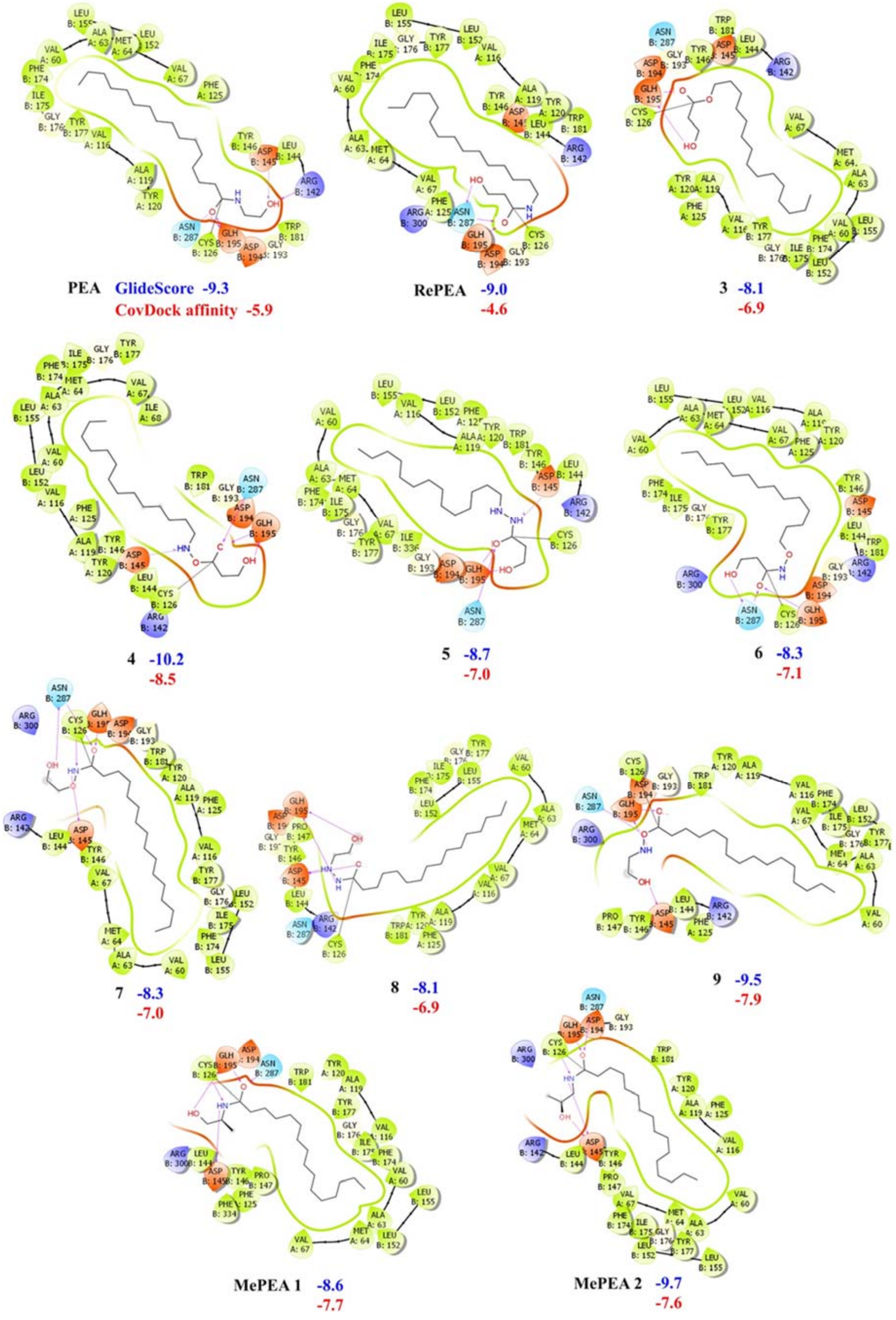

Figure S4. 2D ligand interaction diagrams of **PEA** and analogues with NAAA. GlideScores (kcal/mol, blue values) and CovDock affinities (kcal/mol, red values) are also reported.

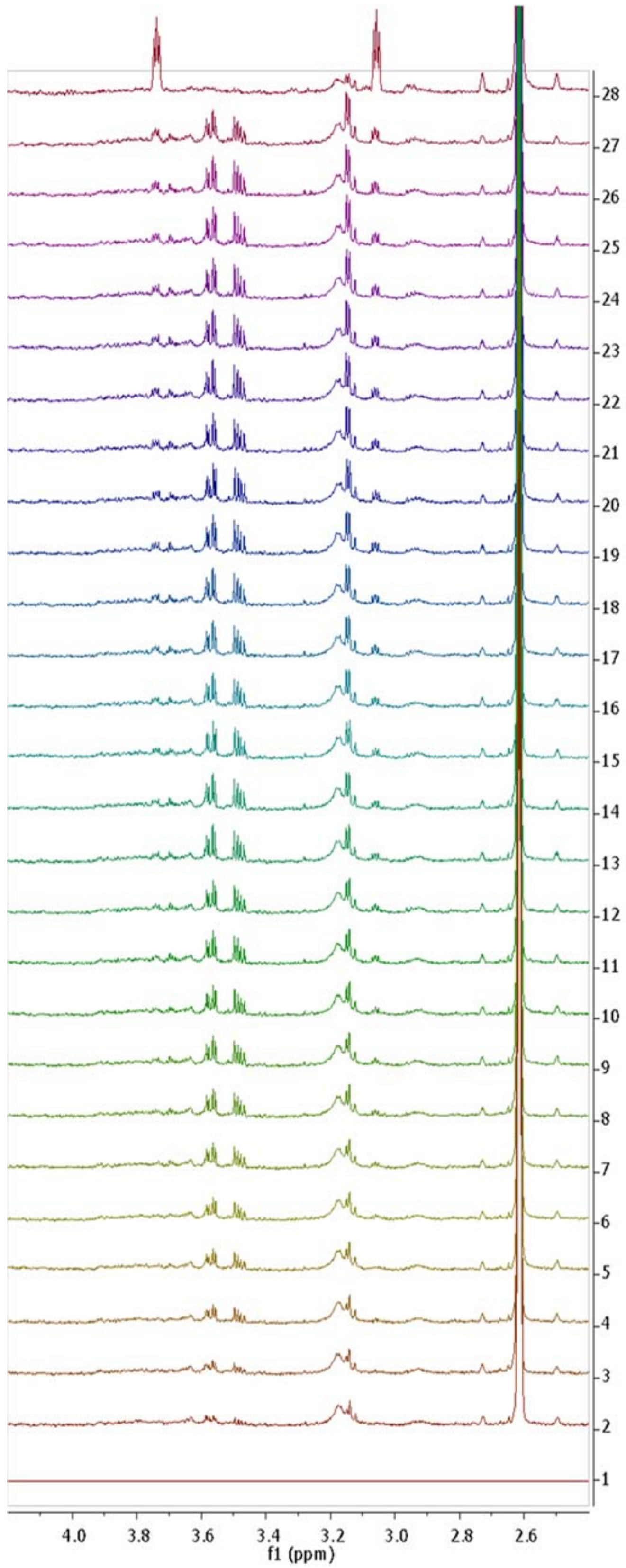

Figure S5A. (**1**) ^{1}H NMR spectrum of a membrane sample dissolved in deuterated PBS, pH 7.4 37 °C. (**2–27**) ^{1}H NMR spectra of the same sample after addition of 1 mM PEA dissolved in d_6-DMSO. Number of scans (NS) = 64; spectrum 2 recorded at t = 0 s after PEA addition, spectra 3–26 recorded in 30 min time intervals over 12 h; spectrum 27 recorded after 24 h. (**28**) ^{1}H NMR spectrum of a membrane sample spiked with 0.1 mM ethanolamine (EA) dissolved in deuterated PBS, 10% d_6-DMSO, pH 7.4 37 °C. PEA NMR resonances are not visible in spectra 2–26 due to PEA interaction in membranes.

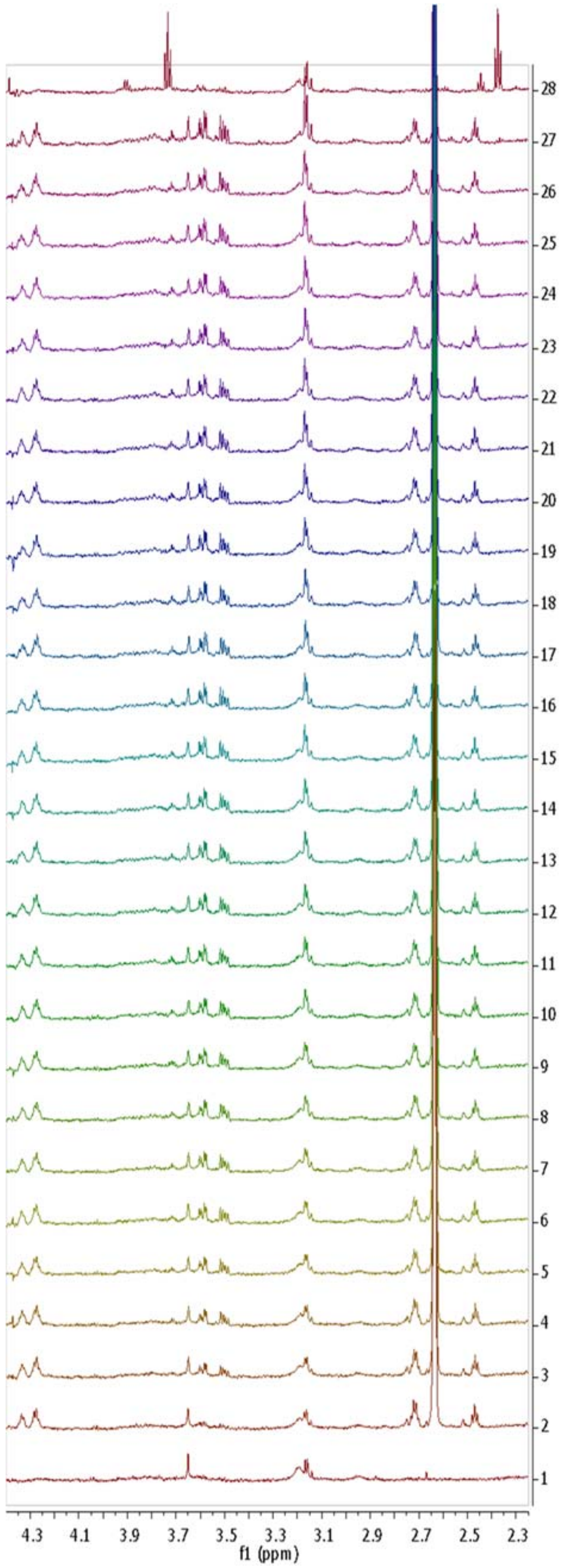

Figure S5B. (**1**) ^1H NMR spectrum of a membrane sample dissolved in deuterated PBS, pH 7.4 37 °C. (**2–27**) ^1H NMR spectra of the same sample after addition of 1 mM retroPEA dissolved in d_6-DMSO. Number of scans (NS) = 64; spectrum 2 recorded at t = 0 s after retroPEA addition, spectra 3-26 recorded in 30 min time intervals over 12 h; spectrum 27 recorded after 24 h. (**28**) ^1H NMR spectrum of a membrane sample spiked with 0.1 mM 3-hydroxipropanoic acid (3-HPA) dissolved in deuterated PBS, 10% d_6-DMSO, pH 7.4 37 °C.

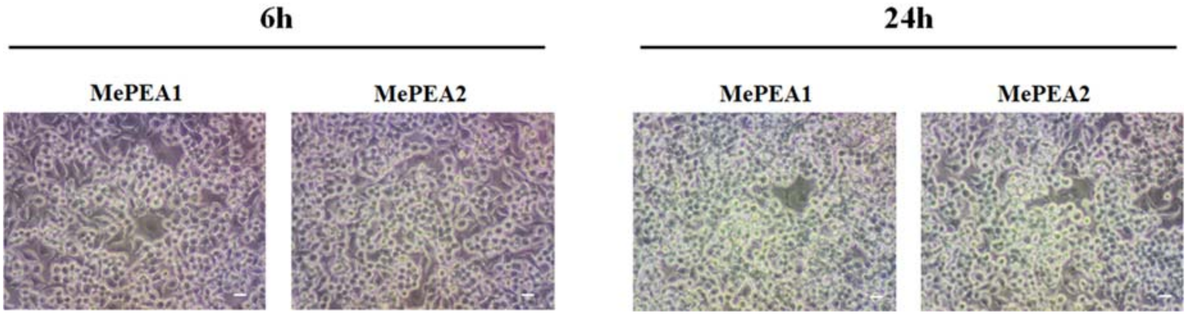

Figure S6. Cellular morphology of N9 cells incubated for 1 h with 100 nM PEA or MePEA1, MePEA2. Then medium was replaced with fresh IMDM supplemented with 5% FBS. After 6- or 24-h optical images were captured with an inverted Olympus CKX41 microscope. Representative images out of at least three separate experiments are shown. Scale bar: 10 μm.

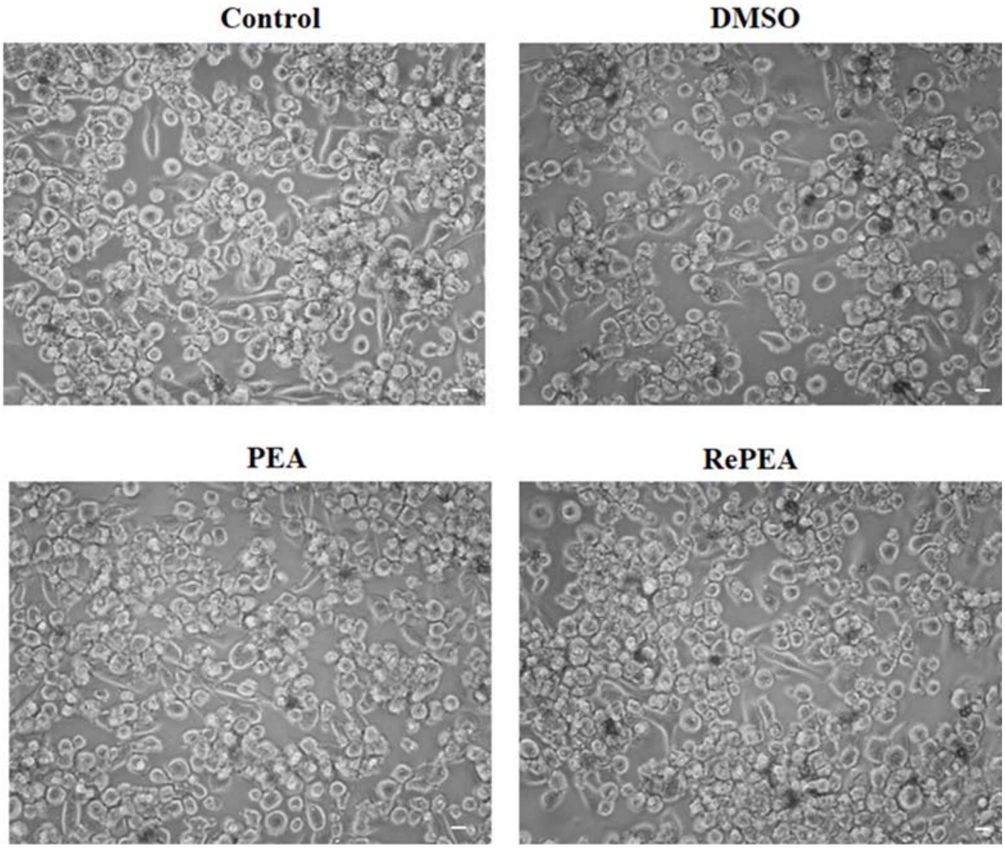

Figure S7. Cellular morphology of PMA-THP-1 X-Blue™ cells incubated for 1 h with 100 nM PEA or RePEA. Then the medium was replaced with fresh RPMI. After 24-h optical images were captured with an inverted Olympus CKX41 microscope. Representative images out of at least three separate experiments are shown. Scale bar: 10 μm.

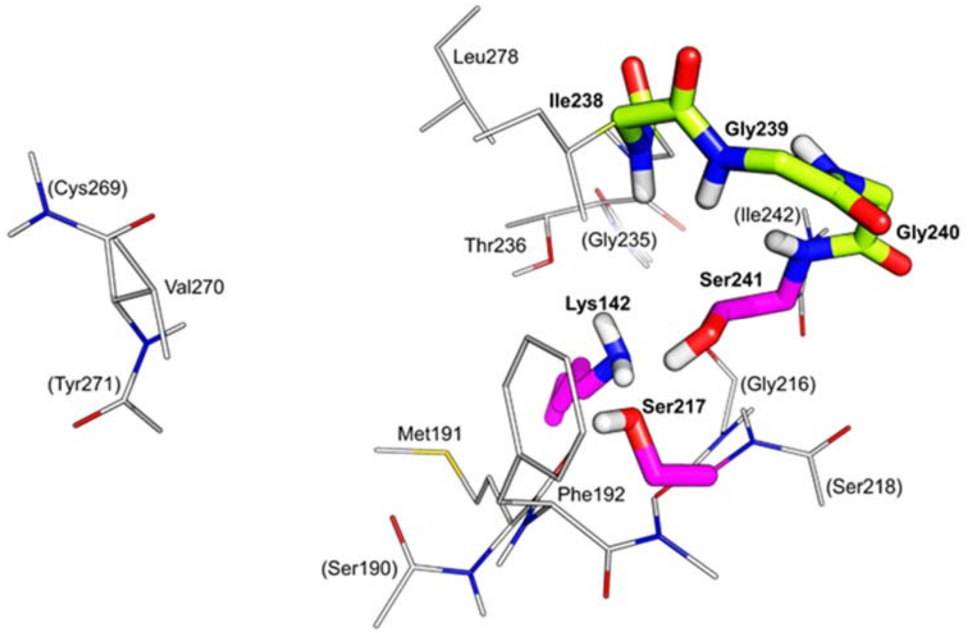

Figure S8. DFT model of FAAH active site. The catalytic triad is colored in purple, while the oxyanion hole in green. The alpha carbons of each residue were fixed at the crystal position during geometry optimizations, in order to avoid artifacts.
